# 青年型非小细胞肺癌治疗研究进展

**DOI:** 10.3779/j.issn.1009-3419.2022.102.48

**Published:** 2022-12-20

**Authors:** 思成 周, 诚 沈, 国卫 车

**Affiliations:** 1 610041 成都，四川大学华西医院胸外科 Department of Thoracic Surgery, West China Hospital, Sichuan University, Chengdu 610041, China; 2 610041 成都，四川大学华西医院肺癌中心 Lung Cancer Center, West China Hospital, Sichuan University, Chengdu 610041, China

**Keywords:** 肺肿瘤, 综合治疗, 青年患者, 临床特点, 预后, Lung neoplasms, Multimodality therapy, Young patients, Clinical characteristics, Prognosis

## Abstract

青年型非小细胞肺癌（non-small cell lung cancer, NSCLC）患者（≤45岁）尽管占比较低，但其有独特的临床病理特征，近年来患病率有上升的趋势。随着肺癌个体化治疗观念的提出，其研究也逐渐受到重视。另外，青年型NSCLC患者在治疗反应和预后等方面与老年肺癌患者有所不同，因此对青年型NSCLC进行研究极具临床意义。本文就青年型NSCLC患者的临床表现、治疗手段和预后等特点作一综述。

## Abstract (en)

Non-small cell lung cancer (NSCLC) young patients (≤45 years old), despite their low prevalence, have unique clinical and pathological features. Its morbidity has been on the rise in recent years. With the concept of individualized lung cancer treatment, related researches are gradually gaining attention. In addition, the treatment response and prognosis in NSCLC young patients are different from older patients, so the study of NSCLC young patients is of great clinical significance. This article reviews the clinical manifestations, treatment and prognosis of NSCLC young patients.

支气管肺癌，简称肺癌，起源于支气管上皮或腺体，结合病理形态和恶性程度可分为小细胞肺癌（small cell lung cancer, SCLC）和非小细胞肺癌（non-small cell lung cancer, NSCLC），其中，NSCLC约占80%^[1]^。肺癌发病率仅次于乳腺癌，位居全球第二位，死亡率位居全球第一位^[2]^；在我国其发病率与死亡率均位居第一位。肺癌患病率随年龄增长而上升，患病高峰期在60岁-64岁。非青年群体发病率是青年群体的30倍-50倍^[1]^。

随着健康意识的提高以及低剂量计算机断层扫描（low dose computed tomography, LDCT）筛查纳入体检项目，肺癌患者中年轻人占比有上升趋势。世界卫生组织（World Health Organization, WHO）将15岁-44岁定义为青年群体，有研究^[3-6]^将15岁-39岁定义为青年群体，同时也有多项研究将年龄介于18岁-40岁的肺癌患者定义为青年型肺癌。综合查阅文献与研究结果后，本文所提及的“青年型肺癌”患者年龄段为18岁-45岁，病理确诊为NSCLC，以便于与既往研究进行比对参考。

青年型NSCLC患者特征与既往流行病学调查显示的肺癌高发群体存在差异，与既往的研究结果存在争议，甚至在预后的预测方面有着较大矛盾。有研究^[7]^认为，青年患者群体在经济条件、受教育程度、基础情况、同分期的情况下，预后好于老年患者；有研究^[8]^则认为，由于青年患者起病隐匿，发病时常为局部晚期或伴有远处转移，且肿瘤细胞分化程度较低，因此整体预后不佳。同时，随着肺癌分子基因层面研究的深入，青年患者的驱动基因改变频率与其他年龄群体的患者相比呈现异质性，有回顾性研究^[9, 10]^揭示了年龄与突变频率的关系。同时青年患者还可能存在独特的驱动基因组突变，如表皮生长因子受体（epidermal growth factor receptor, *EGFR*）/*TP53*。尽管青年患者占比较低，青年型NSCLC很可能是一个相对独立的分组^[11]^，对其进行治疗评估与预后分析时，需要建立更详尽的亚临床组。本文就青年型NSCLC的临床特征、治疗手段及预后和研究展望做一综述。

## 1 青年型NSCLC的临床特征

肺癌作为中国发病率最高的恶性肿瘤，大多数病例属于NSCLC。然而，患者临床特征多变，早期常常症状不典型，而随着疾病的进展，以咳嗽、胸痛、胸闷等症状起病的患者往往病情已较晚，失去了手术机会^[12]^。但一部分患者伴发脑转移时仍未出现临床症状^[13]^。青年型肺癌患者在肺癌患者群体中占比低，统计数据在1%-3%不等^[14]^。尽管一些研究发现被确诊为NSCLC的青年患者比例有增高趋势，但相较于非青年患者，其整体数量仍然偏少，这样的结果意味着可能出现更高的漏诊/误诊率^[15]^。在中国，男、女青年患者肺癌死亡率分别为9‰与5.8‰，分别位居恶性肿瘤死亡率第二位和第三位；美国的调查数据则显示未进入前五^[1, 2]^；肺癌的发病率和死亡率从45岁起陡然上升^[16, 17]^。美国国立综合癌症网络（National Comprehensive Cancer Network, NCCN）2022年版筛查指南建议 > 50岁且有高危因素的人群常规行LDCT筛查^[18]^，青年群体则为“不推荐”，尽管许多青年患者因偶然筛检发现肺部结节，并最终经病理确诊为肺癌。具有高危因素的青年群体能否常规行CT筛查或能否从偶然的CT检查中受益，目前仍无定论。

NSCLC有家族倾向，但家族史很难作为高价值的预测因子纳入NSCLC的管理^[19]^。Lin等^[20]^的回顾性病例对照研究基于中国人群对318例原发性肺癌患者进行了调查，并设置509例患者作为对照。在经过混杂数据处理后发现，有肺癌家族史患者发生肺癌的风险（OR=3.21, *P* < 0.001）高于非肺癌家族史患者（OR=1.79, *P* < 0.001）。亚组分析中则显示一级女性亲属的患病风险往往高于一级男性亲属，提示了肺癌家族史在无吸烟史家庭中的影响。Ang等^[21]^的荟萃分析则提示了被动吸烟、空气污染可能会作为亚洲人群特别是女性高风险的混杂因素，它们可能会在大规模的流行病学调查中对家族倾向研究造成一定干扰。Yu等^[22]^的研究认为有肺癌家族史的非吸烟者可以从消除环境暴露和积极治疗肺部疾病中获益，也提示了遗传因素在青年肺癌群体中的不确定性。目前尚未发现与肺癌遗传明确相关的家族史报道。

随着NSCLC致病机理的深入，NSCLC的精确分类更加依赖分子表型。有研究^[23, 24]^发现了青年型肺癌患者群体部分肺癌基因突变率及突变丰度高于非青年患者群体，且以间变性淋巴瘤激酶（anaplastic lymphoma kinase, *ALK*）重排最典型，*ALK*重排患者中有17.1%合并*EGFR*突变。同期部分研究则对不同的驱动基因进行了相对细化的研究，并揭示了青年群体与非青年群体肺癌驱动基因改变的异质性。Hou等^[25]^前瞻性地对177例 < 45岁肺腺癌患者的病理标本行全基因组测序，发现驱动基因与年龄存在一定的相关性：*ALK*与人类表皮生长因子受体2（human epidermal growth factor receptor 2, *HER2*）基因改变在青年群体中更常见，而*KRAS*、*STK11*与*EGFR*外显子20突变则是相反的趋势，其揭示了年龄 < 45岁NSCLC患者的独特分子谱。此外，并发*EGFR*/*TP53*突变在青年患者中更为普遍（81.6% *vs* 46.8%），这可能部分解释青年肺癌患者对EGFR酪氨酸激酶抑制剂（EGFR tyrosine kinase inhibitors, EGFR-TKIs）治疗反应较差。Kim等^[26]^的研究则聚焦于年龄与*EGFR*突变阳性肺癌的关系，男性患者中，*EGFR*突变发生率与年龄呈负相关（OR=0.76, *P*=0.003）；女性患者中，*EGFR*突变发生率与年龄呈正相关（OR=1.19, *P*=0.02）。总体来讲，青年群体肺癌*EGFR*、*ALK*、*HER2*基因突变率高于非青年群体。但由于驱动基因阳性以腺癌为主，且鳞癌患者年龄中位数较大。同时，二者性别占比有明显差异，鳞癌男性患者占比明显多于腺癌^[27]^。因此，能否依照驱动基因改变频率对青年群体定性仍需要进一步的研究。

随着CT作为常规体检普及后，NSCLC各组织学占比也发生了改变，而青年群体无吸烟史、女性占比高、腺癌为主的流行病学特征相较于其他年龄群体则更为突出^[28, 29]^。一项研究^[2]^认为，拉丁裔与非裔群体罹患NSCLC的概率高于本土白人。驱动基因突变率在族裔间存在差异，Shi等^[30]^的一项前瞻性研究发现了种族与*EGFR*突变频率显著相关（*P* < 0.001）。与白人人群相比，亚洲人*EGFR*突变频率显著较高（51.4%），即使是在既往研究中被认为突变频率较低的男性、吸烟群体。同时，*EGFR*突变阳性患者也存在种族差异，Dahabre等^[31]^的研究显示，在以白人为主的人群中，*EGFR*基因拷贝数的增加与总体生存的提高显著相关（HR=0.70, 95%CI: 0.59-0.82, *P* < 0.001），而东亚患者的生存率则与*EGFR*基因拷贝数无关（HR=1.11, 95%CI: 0.82-1.50, *P*=0.50）。Leidner等^[32]^的研究则发现相较于白人，非裔患者*EGFR*驱动基因处在激活状态的比例较小（2% *vs* 17%, *P*=0.022），证据表明，EGFR通路在不同族裔间存在遗传异质性，不过尚未发现国内开展的相关大型研究。而对于相对常见的*ALK*重排及*KRAS*突变，在族裔间暂未发现显著差异，对于相对少见的*MET*、*ROS1*、*RET*等突变，则仍待进一步的研究。人种基因背景与种族对不同年龄群体发病差异率的影响尚不明晰，需要更多的研究证实。

## 2 青年型NSCLC的治疗手段

### 2.1 手术治疗

青年型NSCLC的手术治疗方式未区别于其他年龄段的患者群体。一般观点认为，手术是能够根治肿瘤的首选手段^[33]^。2022年NCCN指南^[34]^认为：对于一般状况良好、有意愿的患者而言，手术治疗是Tis、T_0-3_N_0-1_分期NSCLC的绝对适应证，是部分T_3-4_N_0-1_分期的相对适应证。青年群体普遍基础疾病少，心肺耐受能力强，手术意愿高。相同分期下可手术的青年患者预后好于非青年患者。Shin等^[35]^的研究证实，接受手术治疗的青年患者群体5年无复发生存率高于高龄患者；且随着时间的推移，其死于非疾病因素的占比显著低于后者。这意味着青年群体接受手术后出现并发症的可能性较低，生活质量较高。对于具有手术指征的青年患者，应积极开展手术治疗。部分回顾性研究发现，到院就医的青年型NSCLC患者分期较晚，确诊为局部晚期或远处转移比例高，但Yoneyama等^[36]^、Chaft等^[37]^的单中心回顾性研究发现，尽管青年患者晚期疾病的比例较高，但青年患者接受手术治疗的总生存期（overall survival, OS）及无进展生存期（progression-free survival, PFS）获益均优于老年患者，非手术治疗的生存获益则劣于手术治疗。因此，青年患者更能受益于积极的多模式治疗。Han的研究^[38]^发现，被确诊为肺癌的青年患者，合并症少，程度轻，体能状态（performance status, PS）评分也普遍较低。无法根治的青年患者能否接受手术为主的综合治疗或姑息性手术，以延长患者OS、提高患者生存质量，目前尚无大规模的临床试验验证^[39]^。

### 2.2 化疗

NSCLC化疗方案相对成熟，随着治疗手段的更新，青年患者难以接受单纯采用铂类为基础的两药方案作为治疗手段^[34]^。此外，NSCLC对化疗敏感程度较低，治疗反应较差，生存获益少^[40, 41]^。青年患者治疗方案往往更激进，对多学科综合治疗手段的接受度也更高^[37]^。当前指南认为，驱动基因阴性的患者不联合免疫抑制剂的化疗方案已不作为常规推荐，其适用范围限制在PS评分高、出现难以耐受免疫相关不良反应及接受姑息治疗的患者。当前指南将免疫治疗手段纳入NSCLC的治疗方案，一线化疗方案联合程序性死亡受体1（programmed cell death 1, PD-1）/程序性死亡配体1（programmed cell death ligand 1, PD-L1）免疫抑制剂可以显著改善驱动基因阴性进展期及晚期肺癌患者的PFS与OS，但获益程度与PD-1相关，并呈现出个体差异^[42]^。然而不幸的是，以上手段对青年群体并未表现出特别的预后受益，其5年生存率与其他患者群体无明显差异^[43]^，NSCLC的内科治疗手段日渐丰富，驱动基因阴性患者预后并未改善，这是当前亟待攻关的难点。

### 2.3 放疗

放疗按照治疗目的分为根治性放疗、辅助放疗和姑息性放疗。根治性放疗适用于拒绝手术或身体情况难以耐受手术治疗的患者群体^[34]^。尽管一些倾向性匹配研究认为特定高龄人群接受根治性放疗及手术治疗的预后存在显著差异，但客观上缺乏青年群体接受根治性放疗的相关研究，因此对于适合手术的青年型NSCLC患者，不做根治性放疗方案的推荐。作为综合治疗组成部分的辅助放化疗，目前没有大样本针对青年型NSCLC的临床研究，这与患者数量少、预后与其他年龄组患者无明显差异有一定关系。而作为新技术的立体定向放疗（stereotactic body radiation therapy, SBRT），Bezjak等^[44]^的研究证明了SBRT在特定人群中的疗效，对于确诊为早期肺癌（T_1-2_N_0_M_0_）但难以耐受或无手术意愿的老年患者，在接受单次11.5 Gy/fx和12.0 Gy/fx放射剂量的两个队列中，其总生存率分别为67.9%（95%CI: 50.4%-80.3%）和72.7%（95%CI: 54.1%-84.8%）；无进展生存率分别为52.2%（95%CI: 35.3%-66.6%）和54.5%（95%CI: 36.3%-69.6%）。Ijsseldijk等^[45]^的荟萃分析则认为对于可手术的NSCLC患者，无论是肺叶切除术还是肺段切除术，其预后均优于SBRT。因此，SBRT能否作为青年型NSCLC的治疗手段仍需要进一步的研究证实。

### 2.4 靶向治疗

靶向治疗是目前青年型NSCLC的前沿方向，对于驱动基因阳性的患者，其治疗获益显著优于传统肿瘤内科治疗方案^[46-48]^。有观点认为，由于青年群体有着更高的驱动基因突变丰度，其应该从靶向治疗中获益更多，但临床研究结果似乎并不完全支持这一观点。Luo等^[49]^的回顾性研究表明除了众所周知的基因突变（如*TP53*和*EGFR*），青年型NSCLC存在少见的潜在肺癌相关基因突变（如*HOXA4*和*MST1*）。肺癌驱动基因拷贝数变异（如*EGFR*和*CDKN2A*）占比多（41.7%, 15/36），肺癌相关的结构变异（如*EML4-ALK*和*KIF5B-RET*）较多（22.2%, 8/36）。青年患者有较高概率存在潜在靶向基因组改变（63.9%, 23/36）。该研究结果有助于描述青年未吸烟患者的详细突变，并对这一独特的NSCLC亚群的分子发病机制提供了参考。青年患者异于非青年患者的分子特征，也许可以解释接受驱动基因阳性患者间的预后差异，Tian等^[50]^的研究中，单中心收集*ALK*融合阳性患者共101例，其中52例为青年患者，伴有罕见的*ALK*融合（包括*CHRNA7-ALK*等）的青年患者对克唑替尼治疗反应良好。均接受一线治疗的青年患者与老年患者的PFS差异有统计学意义（17.5个月 *vs* 9.0个月，*P*=0.048）。然而，同时发生*TP53*突变的青年患者的中位PFS仅为6.2个月。Corrales-Rodríguez等^[51]^的研究分析青年患者驱动基因突变对预后的影响，其结果较为显著，*EGFR*突变阳性青年患者的OS为31.4个月（95%CI: 11.6-51.3），*EGFR*突变阴性则为14.5个月（95%CI: 11.0-17.9）（*P*=0.005）。*ALK*融合阳性OS为9.8个月（95%CI: 3.1-16.5），而*ALK*融合阴性则为5.6个月（95%CI: 3.9-7.3）（*P*=0.315）。*EGFR*和*EML4-ALK*易位频率高于一般人群。Tanaka等^[52]^的研究则证明与无致癌基因改变的老年腺癌患者相比，无致癌基因改变的青年腺癌患者预后明显较差（OS：8.9个月 *vs* 16.4个月，*P* < 0.001），有致癌基因改变的青年患者预后则较好（OS：16.4个月 *vs* 24.9个月，*P* < 0.001）。

### 2.5 消融术

肿瘤消融术作为一种微创技术，为不能接受外科手术治疗的肺癌和肺转移瘤患者提供了一种治疗选择，也被证明是一种安全有效的治疗方法。肺癌的消融治疗常利用射频消融、冷冻消融、微波消融等手段^[53]^。消融术作为手术治疗的替代方案，对于可外科切除的肿瘤特别是文献中认为消融获益较多的≤3 cm的肿瘤组织^[54, 55]^，目前缺少相关临床研究的证实，仅有消融治疗相关的单臂研究。对于分期为早期的青年型肺癌患者，目前仍推荐手术治疗。对于局部晚期或远处转移患者，射频消融能否改善青年患者预后，需要进一步的临床研究予以验证。消融术作为一种重要的非手术治疗手段，局部进展的患者再次行消融治疗仍能获益，不同的消融术之间的优劣仍需要深入研究与探索^[56]^。

## 3 青年型NSCLC的预后

尽管手术方案与术后辅助放化疗及术前新辅助放化疗迭代更新，靶向药物、免疫抑制剂大批上市，NSCLC治疗手段逐渐增多。但相较2000年左右，进展期与终末期NSCLC患者的预后并没有显著的改善^[12]^。有研究认为，青年型NSCLC患者预后劣于非青年群体，Shi等^[57]^的多中心回顾性研究发现，青年组患者中女性（50.84% *vs* 34.85%）、癌症家族史（12.37% *vs* 6.32%）、诊断时晚期（65.46% *vs* 60.77%）和腺癌（65.27% *vs* 61.11%）的比例均高于老年组。两组患者中位OS分别为23.0个月和27.0个月（*P*=0.001），青年群体患者预后差于老年群体。但Yang等^[58]^的研究发现，青年型肺癌的肿瘤学特征更加恶性，其低分化、未分化肿瘤占比较高，中晚期占比高，转移风险高，但肺癌相关死亡率显著低于老年患者。Zhang等^[59]^的研究发现， < 45岁的肺癌患者1年、3年、5年生存率分别为49.87%、26.68%、23.12%。肿瘤原发灶-淋巴结-转移（tumor-node-metastasis, TNM）分期、治疗医院（三级医院与社区医院）、性别和肿瘤组织学被证实为独立预后因素。与上海市其他任何年龄的肺癌患者相比，年轻男性亚组腺癌发生率明显高于对照组（63.77% *vs* 43.19%, *P* < 0.001）。年轻肺癌患者的中位OS与任何年龄的肺癌患者相似，但明显短于中年患者（45岁-60岁）。Xia等^[60]^的研究则发现，青年组与非青年组的肺癌的原发部位及手术治疗方式无统计学差异，T与N分期的组内占比、EGFR突变率也不存在统计学差异；两组患者在诊断为肺癌时的分期分布差异有统计学意义（原位癌/I期/II期占比：青年组38.0% *vs*非青年组40.1%；III期/IV期：青年组62.0% *vs*非青年组52.4%，*P*=0.047）。而总生存率无明显差异。Cimen等^[61]^的单中心回顾性研究则发现大部分青年患者（55/71）诊断时已为晚期肺癌，其中*EGFR*突变阳性13例（18.3%），*ALK*突变阳性13例（18.3%），*ROS*突变阳性2例（2.8%）。手术21例（29.6%），放疗29例（40.8%），化疗48例（67.6%），靶向治疗22例（31.0%）。患者的平均OS为16个月。1年内，41例（57.7%）患者死亡。其研究证明了青年型NSCLC患者独特的分子特征与靶向治疗受益有关。有研究^[62, 63]^发现同分期青年型患者的预后好于老年群体，这与一般状况好、治疗意愿高有关，同时与其区别于老年患者的基因突变丰度有相关性。随着对NSCLC基因层面研究的深入，新一代TKIs如奥西替尼在临床治疗中开始应用^[64]^，*KARS* G12C突变阳性的NSCLC患者中，Sotorasib和Adagrasb单药治疗均可给患者带来临床获益^[65]^；接受*ALK*重排的第三代靶向药物Brigatinib治疗的*ALK*阳性NSCLC患者PFS明显长于接受克唑替尼的患者^[66]^。青年群体独特的驱动基因突变特征，有助于解释治疗预后的差异性，也为更新更优的靶向治疗手段提供了思路。

## 4 展望

NSCLC的治疗手段在最近二十年有了从观念、技术、药物等方面的飞速进展，但青年型NSCLC的治疗相关研究仍不够成熟，目前仍然存在一定的局限性，分析原因主要有以下三点：①青年型NSCLC定义的不清晰及青年组中细分的亚组定义不明确，导致了后期以年龄作为入组标准的相关研究存在差异；②尽管青年型肺癌在近几年占比有升高趋势，但占比相对较低，由于患者一般无明显症状，起病隐匿，导致单中心研究样本较少，现有文献以回顾性研究为主，证据强度不足；③青年型NSCLC治疗手段的研究未体现这一群体的特殊性，而既往的研究对青年型NSCLC的治疗是否有影响、治疗方案的个性化及预后研究的特殊性论证不足，因此，寻求更精准的、个体化的治疗手段，仍然需要高证据强度的临床试验予以验证。
